# Histological, radiological and clinical analysis of the supraspinatus tendon and muscle in rotator cuff tears

**DOI:** 10.1186/s12891-023-06237-9

**Published:** 2023-02-16

**Authors:** Umile Giuseppe Longo, Alessandro Mazzola, Francesco Magrì, Simone Catapano, Sergio De Salvatore, Simone Carotti, Vincenzo Denaro

**Affiliations:** 1 Research Unit of Orthopaedic and Trauma Surgery, Fondazione Policlinico Universitario Campus Bio-Medico, Via Alvaro del Portillo, 200 - 00128 Roma, Italy; 2 Research Unit of Orthopaedic and Trauma Surgery, Department of Medicine and Surgery, Università Campus Bio-Medico Di Roma, Via Alvaro del Portillo, 21 - 00128 Roma, Italy; 3 Unit of Microscopic and Ultrastructural Anatomy, University Campus Bio-Medico, Rome, Italy

**Keywords:** Rotator cuff, Pain, Histology, Atrophy, Fatty infiltration

## Abstract

**Background:**

Macroscopic alterations of the affected rotator cuff (RC) are undoubtedly linked to microscopic changes, but they may underestimate the actual degree of the disease. Moreover, it remains unclear whether preoperative structural RC changes may alter clinical outcomes.

**Methods:**

Supraspinatus tendon and muscle samples were collected from 47 patients undergoing RC surgery. Tendons were evaluated histologically according to the Bonar score; fatty infiltration and muscle atrophy were quantified using a software for biomedical image analysis (ImageJ) in percentage of area affected in the observed muscle section. Preoperative shoulder ROM and pain were evaluated. Radiological muscle atrophy was evaluated with the Tangent Sign and Occupation Ratio; fatty infiltration was assessed according to the Goutallier classification. Correlations between histological, radiological and clinical outcomes were assessed. Statistics were performed using the Spearman correlation coefficient. Intraobserver and interobserver agreement was calculated.

**Results:**

Histopathologic fatty infiltration (*r* = 0.007, *p* = 0.962), muscle atrophy (*r* = 0.003, *p* = 0.984) and the total Bonar score (*r* = 0.157, *p* = 0.292) were not correlated to preoperative shoulder pain. Muscle atrophy showed a significant but weak negative correlation with the preoperative movement of abduction (*r* = -0.344, *p* = 0.018). A significant but weak positive correlation was found between muscle atrophy and the total Bonar score (*r* = 0.352, *p* = 0.015). No correlation between histological and radiological evaluation was found for both fatty infiltration (*r* = 0.099, *p* = 0.510) and muscle atrophy (Tangent Sign: *r* = -0.223, *p* = 0.131; Occupation Ratio: *r* = -0.148, *p* = 0.319). Our histological evaluation showed a modal value of 3 (out of 3) for fatty infiltration and an equal modal value of 2 and 3 (out of 3) for muscle atrophy. In contrast, the modal value of the Goutallier score was 1 (out of 4) and 28 patients out of 47 showed a negative Tangent sign. At histology, intraobserver agreement ranged from 0.59 to 0.81 and interobserver agreement from 0.57 to 0.64. On the MRI intraobserver agreement ranged from 0.57 to 0.71 and interobserver agreement ranged from 0.53 to 0.65.

**Conclusions:**

Microscopic muscle atrophy appeared to negatively correlate with the movement of abduction leading to functional impairment. Shoulder pain did not show any relationship with microscopic changes. Radiological evaluation of the supraspinatus muscle alterations seemed to underestimate the degree of the same abnormalities evaluated at histology.

**Supplementary Information:**

The online version contains supplementary material available at 10.1186/s12891-023-06237-9.

## Introduction

Rotator cuff (RC) disease represents a common source of shoulder pain and dysfunction in adults, although not all patients are symptomatic [1]. The pathogenesis and the natural history of RC tendinopathy is still under debate. It is considered a multifactorial disease [2], with intrinsic (age-related degeneration, genetic predisposition) and extrinsic (micro and macro-traumas) cofactors that contribute to weaken tendons until their rupture [3, 4]. Smoking, hypercholesterolemia and genetics are all proven risk factors for the development of RC tears, influencing the age-related tendon degeneration process [5, 6]. Recently, a gender predisposition for the development of the disease has been theorized [7]. Previous studies have found that, in the early stages of the disease, the supraspinatus tendon may already show the typical histopathologic changes of RC tendinopathy although it appears macroscopically intact [3]. These microscopic tendon alterations promote the progression of pre-existing lesions [8] and predispose the RC to rupture in presence of repeated external traumas [9]. Degenerative changes have been commonly found in many cadaver supraspinatus with no history of shoulder pain [10]. Biochemical changes in tendon matrix are known to predispose a proportion of individuals to chronic rotator cuff tendinitis [11]. In accordance, Hashimoto et al. [12] showed that pathologic tendon changes predate the rupture. The supraspinatus muscle atrophy and fatty infiltration are classic histomorphological changes that develop within muscles after RC tendon tears [8, 13]. They do not improve even after a successful RC repair and the best possibility is the preservation of the preoperative status [8, 13]. Therefore, these findings influence the effectiveness of surgical treatment in terms of functional and symptomatic recovery [14]. Fatty infiltration determines muscle weakness and increases surgical failure rates [15], whereas muscle atrophy is responsible for a reduction of contractile strength [16]. The development of these alterations is probably due to adaptive mechanisms secondary to tendinopathy and they are thought to contribute to the progression of RC lesions [8]. This study aimed to perform a multiparametric analysis of the RC disease, looking for potential relationships between clinical, histological and radiological evaluations. We hypothesize that macroscopic (clinical and radiological) evaluation of the supraspinatus alterations may underestimate the actual (microscopic) degree of shoulder disease. Moreover, it remains unclear whether preoperative structural RC changes may alter clinical outcomes.

## Materials and methods

The Ethics committee of our University approved the present study. Informed consent was obtained from all participants.

### Patients

The study enrolled 77 patients representing a consecutive series of patients treated at the orthopedic department of our institution from November 2017 to May 2019. 9 patients refused to participate to the present study and 21 patients met the exclusion criteria. Overall, we collected full data of 47 patients. All the patients included in the present study undergoing surgical repair had a diagnosis of RC tear. All patients underwent preoperative shoulder magnetic resonance imaging (MRI) scan in the affected side, performed with a 1.5-T unit. Full-thickness rotator cuff tears were measured on the sagittal (at the tuberosities) and coronal images and classified into the numbers of tendons torn by the Senior Author: small tears (< 1 cm of tear and < 1 tendon involved, also called punctiform tear); medium (1–3 cm of tear and 1 tendon involved); large (3–5 cm of tear and 2 tendons involved); massive (> 5 cm of tear and > 2 tendons involved) [17]. Partial-thickness rotator cuff tears were graded by the Senior Author in a binary fashion as either grade 1 (less than 50% torn) or grade 2 (equal or more than 50% torn) and according to the side torn (either articular or bursal) [18, 19]. Conservative management, including nonsteroidal anti-inflammatory drugs, physiotherapy, and rest, failed in all patients, and they continued to experience pain and functional limitation in the affected shoulder. All patients fulfilled the following inclusion criteria: (1) positive RC pathology signs on preoperative examination (at least 1 among Jobe test, Napoleon test, lift-off test, and Patte test) [20], (2) no episodes of shoulder instability, (3) no radiographic evidence of fracture of the glenoid or the tuberosities, (4) MRI evidence of RC tear, (5) RC tear of 1 or more tendons at arthroscopic examination, and (6) no traumatic lesion (lesions involving more than 1 cm of the labrum) of the glenoid labrum or of the capsule at arthroscopic examination. Exclusion criteria were: (1) injections of corticosteroids in the affected shoulder, (2) previous surgical treatment in the shoulder; (3) arthritis of the acromioclavicular or the glenohumeral joint; (4) history of trauma on the affected shoulder.

### Samples collection

**Fig. 1:**
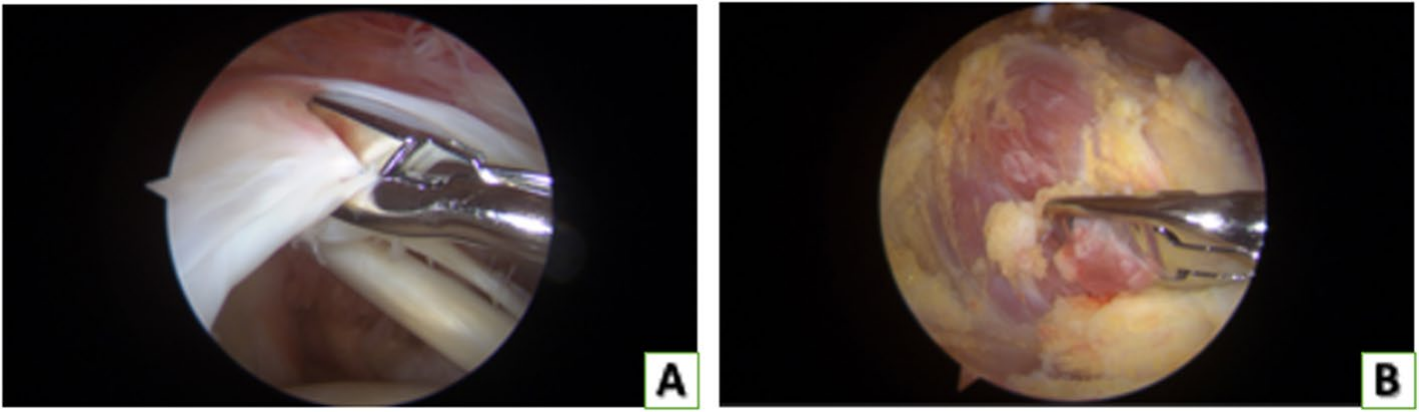
Withdrawal of the supraspinatus tendon and muscle bioptic samples during arthroscopic rotator cuff repair by means of arthroscopic instruments. **A** Withdrawal of a supraspinatus tendon specimen. **B** Withdrawal of a supraspinatus muscle specimen

At arthroscopy, biopsies (about 4 × 4 mm in size) of the supraspinatus tendon (Fig. 1A) and muscle (Fig. 1B) were collected by means of arthroscopic instruments. Tendon specimens were harvested within the arthroscopically intact middle portion of the tendon between the lateral edge of the tendon tear and the muscle–tendon junction. Muscle specimens were collected at the muscle–tendon junction.

### Histopathology and immunohistochemistry

After removing, tissue samples were promptly fixed in buffered formalin 10% at room temperature for 24 h. They were rinsed in phosphate buffered saline (PBS, pH 7,4), dehydrated in an ascending series of alcohol and embedded in paraffin via xylene. Then, 3–5 μm serial sections were cut and processed for Haematoxylin/Eosin, Masson–Goldner Trichrome, Alcian Blu and immunohistochemical stainings. Immunohistochemical analysis was performed using the indirect technique [16, 21]. Sections were deparaffinized and endogenous peroxidase was blocked by incubation in 3% hydrogen peroxide for 5 min at room temperature. The following antibodies were used: mouse monoclonal antibody anti-CD34 (1:100 titre clone QBEnd/10 CM084B; Biocare – Italy) for the evaluation of the microvascular density. After washing with Tris-Buffered saline (TBS), sections were incubated with their primary antibody: CD34 for 2 h at RT. Preparations were then washed three times with PBS, and incubated with their secondary antibody following the kit protocol Dako EnVision Dual Link System-HRP: antimouse (EnVision FLEX LINKER mouse) for 15 min. Sections were then washed three times with TBS and finally incubated in diaminobenzidine (DAB, Dako), for 5 min, followed by haematoxylin counterstaining. Semiquantitative evaluation of immunoreactivity was performed at × 40 magnification in 10 microscopic fields randomly chosen by means of a scientific digital camera (SPOT Insight; Diagnostic Instrument, Inc., Sterling Heights, MI, USA) connected to an Olympus BX-51 light microscope (Olympus, Tokyo, Japan) and elaborated with an Image Analysis System (Delta Sistemi). Negative control slides processed without primary antibodies were included for each staining. Fatty infiltration and muscle atrophy of the supraspinatus muscle were quantified using a software for biomedical image analysis (ImageJ) in percentage of area affected with respect to the whole area of the observed section. For each specimen, fatty infiltration was classified into three grades: grade 1 (< 10%); grade 2 (10–20%); grade 3 (> 20%) of adipose tissue area. Similarly, muscle atrophy was evaluated on sections stained with Masson–Goldner Trichrome, considering three grades of alteration: grade 1 (< 10%); grade 2 (10–20%); grade 3 (> 20%). Semiquantitative analysis of tendinopathy was performed at × 4, × 10, × 20 magnification according to the Bonar score [22, 23]. It includes four parameters: tenocytes, ground substance, collagen, vascularity. Each parameter is scored with a four-point scoring system, in which 0 indicates healthy tissue and 3 highly degenerated tissue. The total Bonar score is comprised between 0 (normal tendon) and 12 (most severe abnormality detectable). For each muscle and tendon specimen, 3 slides were randomly selected and examined with a light microscope. The identification number on each slide was covered with a removable sticker, and each slide was numbered using randomly generated numbers. After 1 of the authors interpreted all the slides once, the stickers were removed, a new sticker was applied, and the slides were renumbered using a new series of randomly generated numbers. The degree of staining of all the slides was reassessed by the same author (an Orthopaedic and Trauma Surgery PhD Student), and the two results were compared. When there was only a 1-grade difference between the two assessments, the highest result was chosen. If an inconsistency (> 1 grade on the scoring system described below) existed between the 2 results, the slides were reassessed with the help of a board certified pathologist (sixth author). Intraobserver and interobserver agreement was calculated.

### Functional assessment

The preoperative range of motion (ROM) of the affected joint was measured. An universal standard goniometer with 1 degree increments was adopted and guidelines for the evaluation of ROMs were followed [24, 25]. Active abduction and external rotation (at 0° and 90° of abduction) of the affected shoulder were recorded preoperatively for each patient. ROM assessment was repeated three times sequentially and arithmetic average was used for statistical analysis. Preoperative shoulder pain evaluation was performed with a visual analogue scale (VAS) pain score (it ranges from 0 cm = “no pain”, to 10 cm = “worst imaginable pain”) [26].

### Radiological assessment

A T2-weighted MRI study's most lateral scan, in which the spine is in touch with the scapular body, is used to analyze the tangent sign [27]. The tangent sign is determined by drawing a line connecting the superior edge of the scapula's spine to the superior edge of the coracoid process. The muscle content should cross superior to the tangent line in healthy tissues. The superior border of the muscle must fell below the tangent line when the tangent sign is positive (showing significant atrophy). When the tangent line and the muscular belly of the supraspinatus intersect, the tangent sign is negative [27].

Oblique sagittal PD-weighted MRI scans were used to calculate the occupation ratio. The boundary of the supraspinatus fossa was closely followed as possible and the supraspinatus muscle outer rim was traced at the most lateral portion of the scapula's Y-view appearance in the oblique sagittal plane. The superior limit of the supraspinatus fossa was the distal clavicle. We used a formula to determine the Occupation Ratio as a measure of muscle volume using the technique provided by Thomazeau et al. [28]. Then, using the formula S1 / S2, the occupation ratio was determined. S1 represents the surface area of the supraspinatus muscle, and S2 represents the surface area of the entire supraspinatus fossa. If the ratio (stage 1) is between 1.00 and 0.60, the muscle is either normal or mildly atrophying. Significant atrophy is indicated by values between 0.60 and 0.40 at the stage 2 level. Stage 3 values below 0.40 indicate severe or moderate atrophy [28].

Using the Goutallier classification as modified by Fuchs et al. [29] on T1-weighted turbo spin-echo sequences, all muscles were evaluated at the most lateral scan on the sagittal view, where the spine was in contact with the scapular body [29] Two additional scans were taken into account for the infraspinatus and subscapularis tendons: an inferior scan at the lowest point of the glenohumeral joint and a superior scan at the level of the lateral attachment of the spine. Five stages are used to categorize changes: Stages 0 and 1 indicate no fat, stage 2 indicates there is more muscle than fat, stage 3 indicates there is an equal amount of fat and muscle, and stage 4 indicates there is more fat than muscle [30].

The MRI images were reassessed by the same author (an Orthopaedic and Trauma Surgery PhD Student), and the two results were compared. The author who performed the evaluation was blinded to clinical findings and to patient’s identities. Intraobserver and interobserver agreement was calculated. When there was only a 1-grade difference between the two assessments, the highest result was chosen. If an inconsistency (> 1 grade on the scoring system described below) existed between the 2 results, the slides were reassessed with the help of a board certified orthopaedist (Senior Author).

### Statistical analysis

Data are expressed as mean and standard deviation (± SD). Since data showed an abnormal distribution, statistics were performed using the Spearman correlation coefficient. Potential correlations were assessed at histology between tendon and muscle alterations (muscle atrophy and fatty infiltration); between histological muscle atrophy and fatty infiltration themselves; between muscle atrophy evaluated at histology and on the MRI; between fatty infiltration evaluated at histology and on the MRI; between histological tendon alterations and clinical outcomes (VAS pain and ROM); between histological muscle atrophy and clinical outcomes (VAS pain and ROM); between fatty infiltration and clinical outcomes (VAS pain and ROM). Only *p* values < 0,05 were considered to be statistically significant. Kappa statistics were used to assess the intraobserver and interobserver agreement.

## Results

The study assessed 47 patients treated at our institution. The mean age of patients was 62.5 years (± 7.4, range 52–78). The rotator cuff tears were preoperatively classified on the MRI as small (< 1 cm) in 5 patients, medium (1- 3 cm) in 12 patients, large (3–5 cm) in 18 patients, and massive (> 5 cm) in 12 patients. Biopsy samples of the supraspinatus tendon and muscle were collected from 19 women (mean age 62.3 ± 8.1) and 28 men (mean age 62.7 ± 6.8) undergoing surgery for RC tears. Histomorphological evaluation of tendon alterations revealed a mean value of the total Bonar scale of 6.5 ± 1.9 (range from 2 to 10) (Table 1).

**Table 1:** Summary of Pathologic scores of the Supraspinatus Tendon. The worst scoring result was used for each situation

Variable	Supraspinatus Tendon				Total tendon pathologic Bonar score	
Grading	0	1	2	3		
Tenocytes	2	9	27	9	Mean	6.5
Ground Substance	10	19	15	3	Median	7
Collagen	0	9	29	9	SD	1.9
Vascularity	15	16	3	13	Range	2–10

**Fig. 2:**
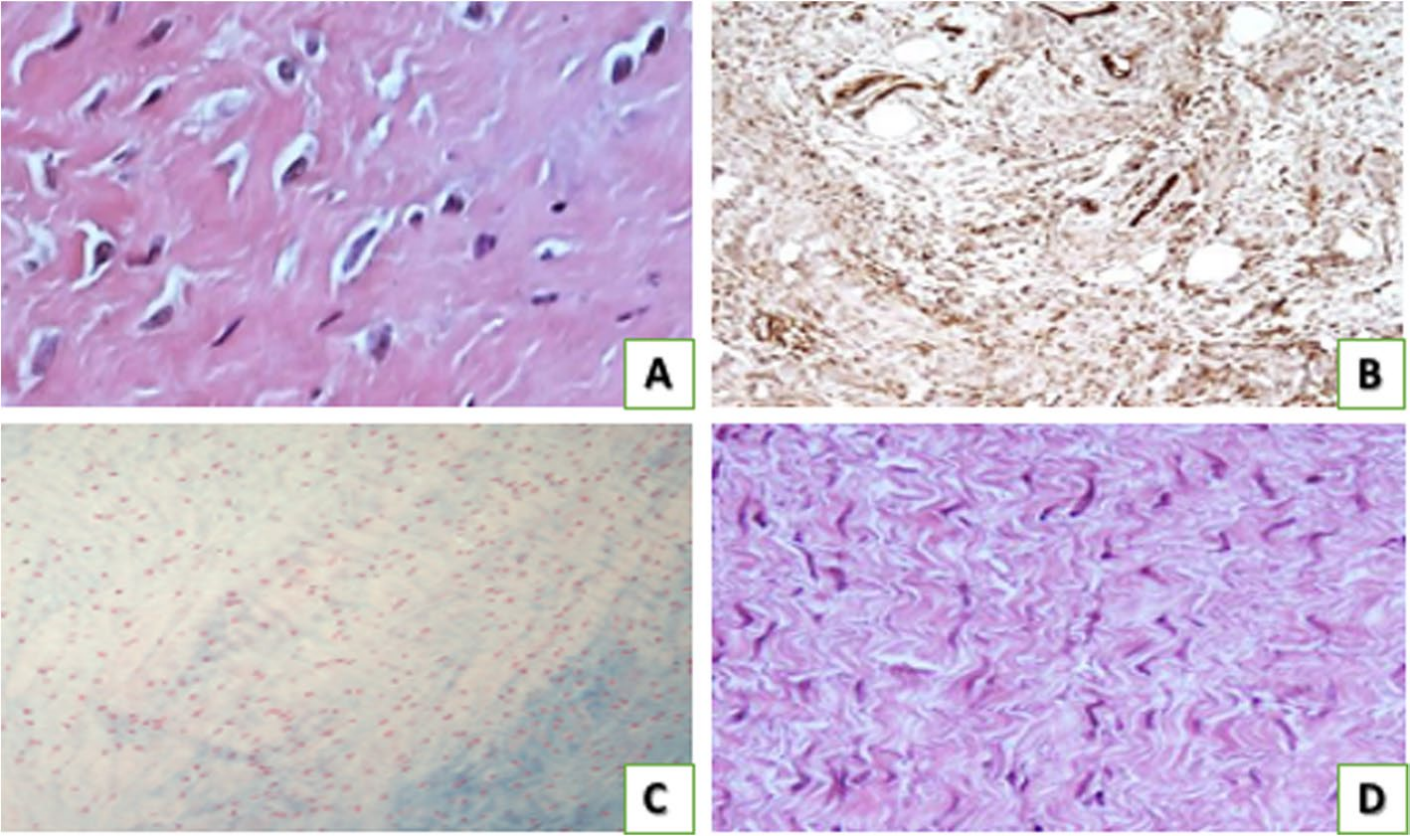
Histomorphological analysis of the supraspinatus tendon alterations according to the Bonar Score. **A** Haematoxylin/Eosin staining showed severe morphological alteration of tenocytes. **B** CD34 immunohistochemical staining shows tendon vascularization. **C** Alcian Blu staining highlights ground substance abnormalities. **D** Haematoxylin/Eosin staining shows disorganization of collagen fibre bundles. Original magnification: × 200 (**A**), × 100 (**B**, **C**), × 150 (**D**)

**Fig. 3:**
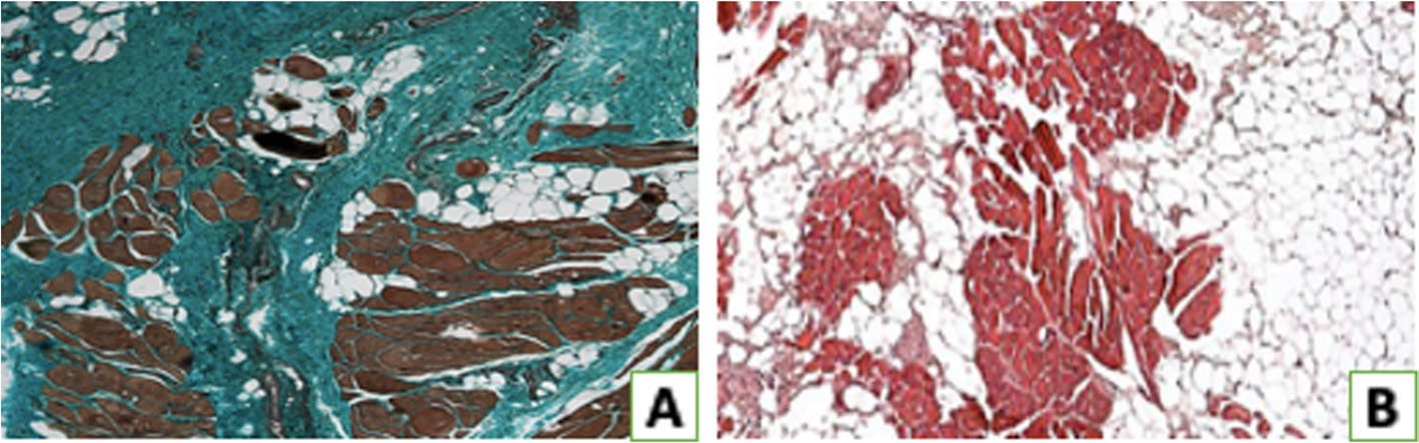
Histomorphological analysis of the supraspinatus muscle alterations. **A** Masson–Goldner Trichrome staining shows fibrous infiltration, responsible for muscle atrophy. **B** Haematoxylin/Eosin staining shows fatty infiltration, surrounding the residual muscle fibers. Original magnification: × 100 (**A**, **B**)

Specimens stained with Haematoxylin / Eosin showed abnormalities of tenocyte morphology in terms of nuclear shape and dimension (Fig. 2A). Grade 2 alteration was the modal value. The immunohistochemical evaluation of tendon vascularity showed occasional pathological clusters of capillaries (Fig. 2B). Grade 1 alteration was the modal value. Alcian blu stainings showed ground substance abnormalities in tendon tissue: intrafascicular and then interfascicular deposits of glycosaminoglycans (GAG) disarraying collagen fibers (Fig. 2C). Grade 1 alteration was the modal value. Masson–Goldner Trichrome and Haematoxylin / Eosin stainings showed disorganization of collagen fibre bundles (Fig. 2D). Grade 2 alteration was the modal value. Masson–Goldner Trichrome stainings allowed the evaluation of the supraspinatus muscle atrophy: amounts of interfibrillar connective tissue mainly composed of disorganized collagen fibers were observed in pathologic tissue samples (Fig. 3A). Grades 2 and 3 were the modal values. Haematoxylin/Eosin stainings of the supraspinatus muscle showed intrafascicular and, in severe cases, perifascicular fatty infiltration [15] (Fig. 3B). Grade 3 level of expression was the modal value (Table 2).

**Table 2:** Summary of Pathologic scores of the Supraspinatus Muscle. The worst scoring result was used for each situation

Variable	Supraspinatus Muscle		
Grading	1	2	3
Fatty Infiltration	15	14	18
Muscle Atrophy	8	19	20

Statistical analysis revealed that histopathological fatty infiltration does not correlate with the preoperative VAS shoulder pain score (*r* = 0.007, *p* = 0.962) (Table 3).

**Table 3:** Summary of ROM assessment and VAS Pain Score. ROM assessment was repeated three times and arithmetic average was used for statistical analysis

Variable	VAS Pain Score	Abduction	External Rotation (at 0° of abduction)	External Rotation (at 90° of abduction)
Mean	5.3	90.4°	41.7°	41°
Median	6	84°	42°	39
SD	2.3	31.7°	21.1°	24.7°
Range	0–9	24–162°	0–68°	97°

Moreover, fatty infiltration did not correlate with preoperative movements of abduction (*r* = 0.139, *p* = 0.351), external rotation at 0° of abduction (*r* = 0.046, *p* = 0.756), external rotation at 90° of abduction (*r* = 0.034, *p* = 0.819). No statistically significant correlation was found between histopathological and radiological fatty infiltration (*r* = 0.099, *p* = 0.510) (Table 4).

**Table 4:** Summary of the population assessed for the Goutallier Score. The worst scoring result was used for each situation

Variable	Goutallier Score				
Grading	0	1	2	3	4
Population	6	24	16	1	0

Histopathological supraspinatus muscle atrophy did not correlate with the VAS shoulder pain score (*r* = 0.003, *p* = 0.984). No significant correlation was found between histopathological supraspinatus muscle atrophy and preoperative movements of external rotation at 0° of abduction (*r* = -0.221, *p* = 0.135), external rotation at 90° of abduction (*r* = -0.096, *p* = 0.522). In contrast, there was a significant but weak negative correlation between histopathological muscle atrophy and the preoperative movement of abduction (*r* = -0.344, *p* = 0.018). No significant correlation was found between the expression of histopathological muscle atrophy and radiological muscle atrophy (Tangent sign: *r* = -0.223, p = 0.131; Occupation Ratio: *r* = 0.122, *p* = 0.413) (Table 5). The total Bonar score did not correlate with the VAS shoulder pain score (*r* = 0.157, *p* = 0.292). No significant correlation was found between the total Bonar score and preoperative movements of abduction (*r* = -0.084, *p* = 0.576), external rotation at 0° of abduction (*r* = -0.281, *p* = 0.055), external rotation at 90° of abduction (*r* = -0.074, *p* = 0.622). At histology, no significant correlation was found between the supraspinatus muscle atrophy and fatty infiltration (*r* = -0.263, *p* = 0.074); no significant correlation was found between fatty infiltration and the total Bonar score (*r* = 0.095, *p* = 0.527); a statistically significant but weak positive correlation was found between the supraspinatus muscle atrophy and the total Bonar score (*r* = 0.352, *p* = 0.015). Using the kappa statistics, at histology intraobserver agreement ranged from 0.59 to 0.81 and interobserver agreement from 0.57 to 0.64. On the MRI intraobserver agreement ranged from 0.57 to 0.71 and interobserver agreement ranged from 0.53 to 0.65 (Table 6).

**Table 5:** Summary of the population assessed for the Thomazeau Stage, the Occupation Ratio and the Tangent Sign

Patient N°	Thomazeau Stage (I-II-III)	Thomazeau Occupation Ratio	Tangent Sign
1	1	0.73	Negative
2	2	0.55	Negative
3	2	0.43	Negative
4	3	0.36	Positive
5	3	0.32	Positive
6	2	0.48	Negative
7	2	0.51	Negative
8	2	0.45	Negative
9	1	0.71	Negative
10	2	0.49	Negative
11	3	0.28	Positive
12	1	0.79	Negative
13	1	0.68	Negative
14	1	0.63	Negative
15	2	0.52	Negative
16	3	0.29	Positive
17	2	0.55	Negative
18	3	0.35	Positive
19	1	0.81	Negative
20	3	0.31	Positive
21	3	0.30	Positive
22	3	0.26	Positive
23	2	0.45	Negative
24	2	0.50	Negative
25	2	0.43	Negative
26	3	0.29	Positive
27	1	0.78	Negative
28	2	0.45	Negative
29	3	0.30	Positive
30	3	0.26	Positive
31	3	0.29	Positive
32	3	0.38	Positive
33	3	0.32	Positive
34	3	0.24	Positive
35	1	0.80	Negative
36	2	0.43	Negative
37	2	0.46	Negative
38	3	0.29	Positive
39	2	0.47	Negative
40	3	0.33	Positive
41	2	0.42	Negative
42	1	0.76	Negative
43	2	0.50	Negative
44	3	0.33	Positive
45	2	0.44	Negative
46	2	0.57	Negative
47	3	0.29	Positive

**Table 6:** Kappa Scores for Each Variable. Kappa Score: 1 indicates a perfect match, and 0 represents no match

Kappa Value									
Tendon and muscle	Tenocytes	Ground Substance	Collagen	Vascularity	Fatty Infiltration	Muscle Atrophy	Goutallier Score	Tangent Sign	Occupation Ratio
Intraobserver agreement	0.72	0.75	0.70	0.59	0.81	0.74	0.62	0.57	0.71
Interobserver agreement	0.59	0.60	0.64	0.57	0.62	0.62	0.54	0.53	0.65

## Discussion

This study has shown that the supraspinatus tendon and muscle of patients with RC tendinopathy present profound histopathological changes, that in part justify the clinical shoulder impairment. However, no correlation was found between histological and radiological evaluation of fatty infiltration and muscle atrophy.

### Histopathology of the degenerated supraspinatus tendon

In healthy tendons, tenocytes and their precursors constitute about 95% of the cellular elements [31]. Tenocytes are flat, tapered cells sparingly distributed among collagen fibrils [31]. They synthesize collagen and all components of the extracellular matrix network [32, 33]. In our samples, tendons showed alterations of tenocyte morphology (in terms of nuclear shape and dimension). It confirms tenocyte changes in tendinopathy with altered tendon cells resembling chondrocytes [31]. Masson–Goldner Trichrome stainings showed disorganization of collagen fibre bundles. The literature indicates that, in healthy tendons, microscopy reveals a hierarchical arrangement of tightly packed, parallel bundles of collagen fibres [34]. Results of the present study confirm the typical modifications of the collagen fibre bundles that become separate and disorganized in patients with RC tendinopathy [35, 36]. In healthy tendons, stainable ground substance (extracellular matrix) is generally absent [34]. In this study, Alcian Blu stainings revealed ground substance abnormalities: intrafascicular and then interfascicular deposits of GAG disarraying collagen fibers. It is known that areas of altered collagen fibre structure and increased interfibrillar ground substance, which has been shown to consist of hydrophilic GAG, correspond with increased signal on the MRI [37] and hypoechogenic regions on ultrasound investigation [38, 39]. The immunohistochemical evaluation of tendon vascularity showed occasional pathological clusters of capillaries. In general, specimens showed low levels of CD34 expression, with a grade 1 alteration mainly observed. As regards the shoulder, vascular insufficiency has been proposed as one of the potential causes of RC disease [40, 41]. In particular, has been shown that diminished vascular supply is associated with degenerative RC lesions [42, 43]. These results are consistent with previous studies in which blood flow and vascular density in the affected supraspinatus tendon appeared to be significantly lower if compared with the normal tendon [22, 44, 45].

### Histopathology of the degenerated supraspinatus muscle

Persistent atrophy of muscle fibers and an accumulation of fat, commonly referred to as fatty infiltration, generally occur in patients with chronic and massive RC tears [30]. The etiology of fatty infiltration and function of the residual rotator cuff musculature have not been well characterized yet [46]. Current surgical treatments are unable to alter or reverse the progression of fatty infiltration and are associated with poor functional outcomes in these patients [47, 48]. Our Haematoxylin / Eosin stainings of the supraspinatus muscle showed high levels of fatty infiltration, with intrafascicular and, in severe cases, perifascicular localization. Moreover, Masson–Goldner Trichrome stainings allowed the evaluation of the supraspinatus muscle atrophy: amounts of interfibrillar connective tissue mainly composed of disorganized collagen fibers were observed in pathologic tissue samples.

### Reliability of histopathological and radiological interpretation

In this study, each specimen was assessed twice with the help of a board certified pathologist. Also the radiological evaluations were repeated twice with the help of a board certified orthopaedist. The kappa statistics showed that the intraobserver and interobserver agreement of blinded assessment for the various tendon and muscle scoring systems is acceptable. Whether these methods could be implemented in clinical practice or in research studies is open to discussion [49].

### Clinical implications

A clinically relevant finding is that, microscopically, the quantification of degree of tendon alteration confirmed to be strictly linked to the degree of muscle atrophy. This finding remarks the importance of an early diagnosis for RC tendinopathy: the activation of molecular patterns in the adjacent muscle, consequent to tendon tears, is responsible for a progressive and irreversible muscular involution [13, 50]. Despite fatty infiltration and muscle atrophy are classic morphologic changes in chronic RC tears, how these two alterations develop and what are the molecular driving patterns remains unclear [8, 13, 50]. Results of the present study did not show any reciprocal influence of these two abnormalities on muscles. It can be speculated that, although they are both features of the same disease, they may be driven by different pathogenic triggers. Our evidence that tendon alterations were linked to the degree of muscle atrophy but independent from fatty infiltration seems to be in line with this theory. This is in accordance with previous studies where, on patients who had undergone successful RC repair, no relationships were found in the postoperative evolution of muscle atrophy and fatty infiltration [14].

The association of structural characteristics with pain and function in patients with RC tears is still debated [6, 51, 52]. This study reported that microscopic tendon alterations, fatty infiltration and muscle atrophy do not correlate with shoulder pain. Theses results are in line with the current literature: previous studies have shown that morphological tear severity is not associated with patient-reported shoulder pain [6, 51, 53]. In contrast, other authors highlighted that biomechanical, epidemiological and psychological factors may influence shoulder pain patient perception [6, 51, 53].

The supraspinatus compresses, abducts and provides a small external rotation torque to the glenohumeral joint [54]. Studies have demonstrated the contribution of the supraspinatus to abduction [55, 56]. Furthermore, this muscle provides weak external rotation regardless of abduction angle, although it appears to be a more effective external rotator at smaller abduction angles [57]. In the present study, the active preoperative ROM of the shoulder was recorded. Results showed a statistically significant correlation between histopathological muscle atrophy and the preoperative movement of abduction. The correlation is negative, it means that the more muscle atrophy increases, the more the abduction movement is impaired. We have harvested only supraspinatus samples, in patients with rotator cuff tendinopathy. When left untreated, supraspinatus muscle atrophy and fatty infiltration typically progress over time from the moment of tendon detachment [58]. Mechanical detachment of the tendon is the primarily responsible for fatty infiltration and rotator cuff atrophy. Moreover, time from onset of symptoms to diagnosis is associated with progression of these two abnormalities [58]. Muscle atrophy is believed to be irreversible and contributes to poor functional outcomes after tendon repair [8, 13, 50]. It can be assumed that higher levels of muscle atrophy belong to older lesions, with a more heterogeneous impairment of the rotator cuff components. Accordingly, results of the present study have found a negative correlation between supraspinatus muscle atrophy and loss of abduction. These findings suggest a major role of the muscle atrophy, rather than tendon degeneration and fatty infiltration, in impairing the supraspinatus clinical function. Surprisingly, histological and radiological fatty infiltration did not show any significant correlation. The same occurred for muscle atrophy. This result may be in part attributable to the spareness of our population. Our histological evaluation showed a modal value of 3 (out of 3) for fatty infiltration and an equal modal value of 2 and 3 (out of 3) for muscle atrophy. In contrast, the modal value of the Goutallier score was 1 (out of 4) and 28 patients out of 47 showed a negative Tangent sign. These discrepancies in grading the same alterations by means of different techniques suggest that macroscopic (clinical and radiological) evaluation of the shoulder may underestimate the actual degree of disease. These may lead to late diagnosis increasing failure rates [59].

### Limitations of the present study

We are fully aware of the limitations of this study. For example, a relatively small sample size did not allow us to perform large-scale statistical analyses. Moreover, we did not have any control group of unaffected supraspinatus muscle and tendon samples. The ideal control should not have any shoulder abnormalities and systemic disease. due to the lack of a control group we were unable to compare our findings with normal healthy tissue. However, for ethical and practical reasons, no alternatives were possible, as it is impossible in our setting to take surgical biopsy specimens from healthy individuals. When interpreting the results of this study, it should be pointed out that we used only 3 staining methods and 1 immunohistochemical analysis. Obviously, more advanced histochemical and immunohistochemical techniques and electron microscopy analysis would allow better estimation of the supraspinatus abnormalities. However, stainings employed in the present study are widely available, cost-effective, and require little technical ability. We harvested muscle and tendon specimens from the supraspinatus myo-tendinous junction: there is still no evidence that the status of the myo-tendinous junction is the same of the muscle or tendon core. Furthermore, in the present study, 2D histology with 2D imaging were correlated: It is known that the muscle status changes over its course from medial to lateral and proximal to distal. It may have affected the assessment of the actual degree of the supraspinatus muscle disease. In the current literature that we know of there is not a validated semiquantitative histopathologic classification of the supraspinatus muscle atrophy or fatty infiltration. We are aware that the classification system adopted in the present study may have some limitations. Moreover, despite their statistically significance, the two correlations of the present study are weak: further studies with larger sample size are needed in order to confirm these results. On the MRI, the tangent sign was used to judge supraspinatus muscle atrophy [27]: we are aware of the high risk of false positives because of the retraction from larger rotator cuff tears when using a relatively lateral sagittal view.

Referring to the radiological evaluation, given the fact that radiologists and surgeons do not agree well when analyzing images [60], a potential limitation of the present study is the lack of a Board Certified Radiologist among authors. A comparison between Orthopaedists and Radiologists in the assessment of MRI findings would have improved the study quality.

Finally, we are not aware of the level of rotator cuff tendon and muscle degeneration in the general Italian adult population. We are not aware of any study detailing the histological appearance of rotator cuff tendon degeneration in this population.

## Conclusions

Microscopic tendon alterations occur together with muscle alterations in patients with RC tendinopathy. Muscle atrophy is a direct consequence of tendon detachment and an early diagnosis is mandatory in order to prevent irreversible changes. Microscopic alterations are intrinsically linked to clinical evidence of disease: findings of the present study suggest a major role of the muscle atrophy, rather than tendon degeneration and fatty infiltration, in impairing the supraspinatus clinical function. Shoulder pain seems to be independent from morphological tendon and muscle alterations and further studies are required in order to investigate its triggers. Radiological evaluation of the supraspinatus muscle may be not so accurate in determining the actual stage of the disease, increasing the risk of late diagnoses.

## Supplementary Information

**Additional file 1.**

## Data Availability

The dataset supporting the conclusions of this article is included within the article.

## Abbreviations

RC: Rotator cuff
ROM: Range of motion
MRI: Magnetic resonance imaging
PBS: Phosphate buffered saline
TBS: Tris-Buffered saline
DAB: Diaminobenzidine
VAS: Visual analogue scale
SD: Standard deviation
GAG: Glycosaminoglycans
