# Supplementary material for: Histological, radiological and clinical analysis of the supraspinatus tendon and muscle in rotator cuff tears
Source: BMC Musculoskelet Disord. 2023 Feb 16;24:127. doi: 10.1186/s12891-023-06237-9 (PMC9933395; doi:10.1186/s12891-023-06237-9)
Supplement: Supplementary file 1 — Additional file 1. [file 12891_2023_6237_MOESM1_ESM.docx]

1. **Table: Thomazeau Stage and Occupational Ratio of patients included in the study:**

| **Patient N°** | **Thomazeau Stage (I-II-III)** | **Thomazeau Occupation Ratio** |
| --- | --- | --- |
| 1 | 1 | 0.73 |
| 2 | 2 | 0.55 |
| 3 | 2 | 0.43 |
| 4 | 3 | 0.36 |
| 5 | 3 | 0.32 |
| 6 | 2 | 0.48 |
| 7 | 2 | 0.51 |
| 8 | 2 | 0.45 |
| 9 | 1 | 0.71 |
| 10 | 2 | 0.49 |
| 11 | 3 | 0.28 |
| 12 | 1 | 0.79 |
| 13 | 1 | 0.68 |
| 14 | 1 | 0.63 |
| 15 | 2 | 0.52 |
| 16 | 3 | 0.29 |
| 17 | 2 | 0.55 |
| 18 | 3 | 0.35 |
| 19 | 1 | 0.81 |
| 20 | 3 | 0.31 |
| 21 | 3 | 0.30 |
| 22 | 3 | 0.26 |
| 23 | 2 | 0.45 |
| 24 | 2 | 0.50 |
| 25 | 2 | 0.43 |
| 26 | 3 | 0.29 |
| 27 | 1 | 0.78 |
| 28 | 2 | 0.45 |
| 29 | 3 | 0.30 |
| 30 | 3 | 0.26 |
| 31 | 3 | 0.29 |
| 32 | 3 | 0.38 |
| 33 | 3 | 0.32 |
| 34 | 3 | 0.24 |
| 35 | 1 | 0.80 |
| 36 | 2 | 0.43 |
| 37 | 2 | 0.46 |
| 38 | 3 | 0.29 |
| 39 | 2 | 0.47 |
| 40 | 3 | 0.33 |
| 41 | 2 | 0.42 |
| 42 | 1 | 0.76 |
| 43 | 2 | 0.50 |
| 44 | 3 | 0.33 |
| 45 | 2 | 0.44 |
| 46 | 2 | 0.57 |
| 47 | 3 | 0.29 |

1. **Selection process flow diagram:**

Patients enrolled in the study

(n=77)

Patients who refused to partecipate

(n=9)

Patients meeting the exclusion criteria (n = 21)

- Injections of corticosteroids in the affected shoulder (n = 8)
- Previous surgical treatment in the shoulder (n = 4)
- Arthritis of the acromioclavicular or the glenohumeral joint (n = 4)
- history of trauma on the affected shoulder (n = 5)

Patients involved in the study

(n=68)

Patients included in the study

(n=47)
